# Cebpb Regulates Skeletal Stem Cell Osteogenic Differentiation and Fracture Healing via the WNT/*β*-Catenin Pathway

**DOI:** 10.1155/2022/2091615

**Published:** 2022-07-18

**Authors:** Jiansong Wang, Chensong Yang, Fanyu Kong, Zhi Zhang, Shengchao Ji, Guixin Sun

**Affiliations:** Department of Orthopedic Trauma, Shanghai East Hospital, School of Medicine, Tongji University, Shanghai 200120, China

## Abstract

Fracture is the most common traumatic organ injury, and fracture nonunion is a critical clinical challenge. The research on the mechanisms of skeletal stem cell (SSC) differentiation and fracture healing may help develop new treatment strategies and improve the prognosis of patients at high risk of nonunion. Bioinformatic analysis of scRNA-seq data of mouse SSCs and mouse osteoprogenitors was applied to discover major transcription factors for the regulation of SSC differentiation. FACS was used to isolate SSCs prospectively. The expression of Cebpb, osteogenesis-related genes (Runx2, Sp7, and Bglap2), and markers for Notch, Hedgehog, MAPK, BMP2/SMAD, and WNT/*β*-catenin signaling pathways (Hes1, Gli1, p-Erk1/2, p-Smad1/5/9, and *β*-catenin) were detected in SSCs with qPCR or western blot, respectively. Alkaline phosphatase assay and alizarin red S staining were used to illustrate the osteogenic differentiation ability of SSCs in vitro. A WNT inhibitor, IWR-1, was further used to explore the mechanism of WNT signaling in the differentiation of SSCs. Micro-CT, mechanical testing, and immunohistochemistry of osteogenic and chondrogenic proteins (Sp7 and Col2*α*1) were used to demonstrate the capacity of Cebpb knockdown in promoting fracture healing in a monocortical defect model. We found that Cebpb was the crucial transcription factor regulating SSC differentiation. Inhibiting Cebpb in SSCs enhanced the expression of active *β*-catenin to promote the expression of WNT target genes, thus facilitating the osteogenic differentiation of SSCs. Bone mass, mechanical properties, and osteogenic protein expression were also increased in the Cebpb inhibition group compared to the group without Cebpb inhibition. Collectively, our results proved that Cebpb knockdown promotes SSC osteogenic differentiation and fracture healing via the WNT/*β*-catenin signaling pathway.

## 1. Introduction

Fracture is the most common traumatic organ injury, and the incidence rate rises rapidly with population aging [1–3]. Scarless healing can be achieved in most cases, whereas 5%-10% of patients show delayed bone healing or nonunion, which is a critical clinical challenge [2, 4]. For each fracture complication, the treatment cost will increase by 12,000 US dollars, and the occurrence of nonunion may double the medical cost [1, 5]. The most common locations of nonunion are the scaphoid, tibia plus fibula, and femur, which play a pivotal role in weight-bearing and flexible movement [4]. Promoting fracture healing and treating nonunion have benefits in reducing medical expenses and improving quality of life.

Many strategies, including surgery, electromagnetic field stimulation, low-intensity pulsed ultrasound stimulation, parathyroid hormone, and osteoinductive factors, have been developed to promote fracture healing and treat nonunion, but there is still no consensus on the treatment [6, 7]. Osteoinductive and morphogenetic factors have been considered as the most promising biological enhancement and studied widely. The FDA has approved three growth factors for bone regeneration, including recombinant human bone morphogenic protein-2 (rhBMP2, brand name: Infuse® Bone Graft), recombinant human bone morphogenic protein-7 (rhBMP7, brand name: OP-1 Putty), and recombinant human platelet-derived growth factor-BB (rhPDGF-BB, brand name: AUGMENT® Bone Graft). However, these growth factors have some side effects. OP-1 Putty has withdrawn from the market. Infuse® Bone Graft has complications such as soft tissue swelling, ectopic bone formation, and tumor formation. AUGMENT® Bone Graft is safer; however, it is only approved for hindfoot and ankle arthrodesis [8]. The research on the mechanisms of osteoblast differentiation and fracture healing may help develop new treatment strategies and improve the prognosis of patients at high risk of nonunion.

Cell therapy has made important progress in the field of regenerative medicine and has also been extensively studied in bone regeneration [7, 9]. Mesenchymal stem cells (MSCs) were studied as seed cells to accelerate fracture healing and achieved important advances for many years [10]. However, the heterogeneity of nonskeletal tissue-derived MSCs and their limited differentiation capacity in vivo restrict their further application. In recent years, people have turned to study skeletal tissue-derived stromal cells with specific osteogenic and chondrogenic differentiation capabilities in vivo, which are called skeletal stem cells (SSCs) [11–18]. SSCs are defined as slow-cycling stem cell that exists in multiple locations in the bone. Under stimulation, they coordinate and supply various types of cells, including chondrocytes and osteoblasts, to help bone growth, bone remodeling, and bone repair [11, 13, 19]. Prospective isolation of stem cells can facilitate the rapid progress in understanding their biology. SSCs and its differentiated cells of different lineages have been identified by prospective isolation methods, among which CD45^−^Tie2^−^Ter119^−^CD51^+^6C3^−^Thy^−^CD105^−^ cells in bone tissues are considered as SSCs [15, 16, 19]. Studies have shown that fracture can promote the expansion and differentiation of SSCs and osteoprogenitors (OPs). Impaired expansion and osteogenic differentiation of SSCs may lead to delayed fracture healing and nonunion [15]. Therefore, factors that promote osteogenic differentiation of SSCs may help accelerate fracture healing and treat nonunion.

Transcription factors (TFs) are proteins that can bind DNA in a sequence-specific manner and regulate transcription [20]. Many TFs act as master regulators, controlling the process of cell type decision and development mode. TFs can promote differentiation and dedifferentiation of bone marrow stromal cells (BMSCs) [20, 21]. For example, the introduction of four TFs (Oct4, Sox2, c-Myc, and Klf4) into mouse fibroblasts can induce them to transform into induced pluripotent stem cells (iPSCs) [22]. Runt-related transcription factor 2 (Runx2) and Sp transcription factor 7 (Sp7) are critical regulators in the differentiation of osteocytes and markers for osteoprogenitors and preosteoblasts, respectively [23]. Exploring TFs playing critical roles in the differentiation of SSCs may help cell therapy for nonunion.

In this study, by comprehensively analyzing the single-cell sequencing (scRNA-seq) data of mouse SSCs (mSSCs) and mouse OPs (mOPs), we explored the TFs that play a key role in the process of SSC differentiation. We confirmed the critical role of Cebpb (CCAAT/enhancer-binding protein-*β*) in SSC differentiation and fracture healing through differentiation experiments in vitro and transplantation in vivo. Our research also uncovers the mechanisms underlying SSC differentiation. Our findings are useful for understanding the mechanisms regulating SSC differentiation and developing cell therapy for nonunion.

## 2. Materials and Methods

### 2.1. Data Collection, Preprocessing, and Integration

Keywords “skeletal stem cell” and “differentiation” were used to search the GEO website (https://www.ncbi.nlm.nih.gov/geo/). Four datasets were found, and GSE142873 was chosen as it has two different stages of mSSC differentiation. GSE142873 was scRNA-seq data and included 37 mSSC and 70 mOP samples. The normalization and preprocessing were executed by R package Seurat [24] (version 4.0). Cell cluster division was based on the published article [16]. Dimensionality reduction analysis was performed by the RunTSNE function. The FindAllMarkers function in the Seurat package was used to calculate the markers of each cluster. Markers that met the following criteria were chosen: |log2 fold change (FC)| ≥ 0.25 and *p* value ≤ 0.05. The chosen markers were applied for Gene Ontology (GO) biological process (BP) enrichment analysis by the clusterProfiler package [25] (version 3.14). Significance was demarcated by Benjamini–Hochberg (BH) adjusted *p* < 0.01.

The analysis of single-cell gene regulatory network was performed using the SCENIC package [26] (version 1.02). Briefly, the scaled expression matrix was used to build the initial gene coexpression networks by GENIE3 algorithm. The regulon data was then analyzed using the RcisTarget algorithm to create TF motifs using mm10-tss-centered-10 kb database. The regulon activity scores were calculated with AUC by the AUCell algorithm. Significant regulons enriched in different clusters were calculated by Wilcoxon test. Pheatmap (version 1.0.12) was used to draw the heat map of significant cluster markers and TFs

### 2.2. Isolation and Culture of mSSCs

Fluorescence-activated cell sorting (FACS) was used to isolate mSSCs as previously reported [16, 27]. Briefly, bones of postnatal 3-day (P3) C57/BL6 mice were dissected after euthanasia and digested mechanically with scissors and a mortar under a stereomicroscope. Subsequently, skeletal tissues were incubated with digestive enzymes, which are composed of type I collagenase (2 mg/ml) and nuclease (1 mg/ml) at 37°C for 40 min under constant agitation. Total dissociated cells were filtered through 40 *μ*m nylon mesh, pelleted at 200*g* at 4°C, resuspended in staining buffer, blocked with rat IgG, and stained with fluorochrome-conjugated antibodies against CD45 (BD Biosciences, Franklin Lake, NJ, USA), Tie2 (BD Biosciences), TER119 (BD Biosciences), CD51 (BD Biosciences), Thy (BD Biosciences), 6C3 (Miltenyi, Cologne, Germany), and CD105 (Miltenyi) for purification by FACS. 7-AAD was used to label and exclude any dead cells. FACS was performed on Beckman MoFlo Astrios EQ system and analyzed using FlowJo (version 10).

mSSCs were cultured in complete medium, which consists of low-glucose DMEM, 10% fetal bovine serum, 100 U/ml penicillin, 100 U/ml streptomycin, and 2 mM L-glutamine. The culture environment is 37°C with 5% CO_2_. The medium was refreshed every 3 days. When confluence reached approximately 80%, cells were detached and passaged. Cells at passage 3 were used for all experiments.

### 2.3. Plasmid Construction, Production of Lentivirus, and Transfection

To generate the Cebpb-knockdown cells, lentivirus containing short hairpin RNA (shRNA) targeting Cebpb was constructed. The target and control short hairpin sequences are as follows: shRNA-1, CTGACGCAACACACGTGTAACCTCGAGGTTACACGTGTGTTGCGTCAG; shRNA-2, CACCCTGCGGAACTTGTTCAACTCGAGTTGAACAAGTTCCGCAGGGTG; and control shRNA, CCTAAGGTTAAGTCGCCCTCGCTCGAGCGAGGGCGACTTAACCTTAGG. The pLKO.1-puro plasmid was linearized using AgeI and EcoRI restriction enzymes and purified on 1% agarose gel. Target sequences and linearized plasmids were ligated with a T4 DNA ligase according to the manufacturer's introduction (Sangon Biotech, Shanghai, China).

Lentivirus containing full-length Cebpb cDNA was constructed to generate Cebpb-overexpression cells. The primers for PCR amplification and seamless cloning of the gene-coding regions were as follows: 5′ primer, CATAGAAGATTCTAGAGCCGCCATGCACCGCCTGCTG, 3′ primer, ATTTAAATTCGAATTCCTAGCAGTGGCCCGCC. In the forward primer sequence of the control vector, ATG was changed to TAG to make it not translated. Kozak sequences (GCCGCC) were inserted before the start codons to enhance translational efficiency. The pCDH-CMV-MCS-EF1-puro vector was linearized using XbaI and EcoRI restriction enzymes and purified on 1% agarose gel. The cDNA was amplified by PCR and cloned into pCDH-CMV-MCS-EF1-puro vector by seamless cloning according to the manufacturer's introduction (Sangon Biotech, Shanghai, China).

The lentiviruses were produced by transfecting transfer plasmid, packaging plasmid (psPAX2), and envelop plasmid (pMD2.G) into 293T cells using the Lipo3000. Supernatants were harvested at 48 and 72 h after transfection. Lentiviral particles were concentrated using PEG6000 precipitation combined with differential centrifugal method.

For transfection, 2 × 10^5^ cells were seeded into 6-well plates and incubated with lentiviruses and 5 *μ*g/ml polybrene in the incubator for 24 h. After 48 h, puromycin was added into the medium to select mSSCs stably expressing the puromycin resistance gene.

### 2.4. CCK8 Assay

CCK8 assay was chosen to study the proliferation of mSSCs. Briefly, mSSCs were cultured in 96-well plates at 5 × 10^3^ cells/well. After culturing for 1 day, CCK8 (10 *μ*l/well, Dojindo, Japan) was added into each well and incubated for 2 h at 37°C. The absorbance value was measured using a microplate reader (BioTek, Vermont, USA) at 450 nm. Growth curve was drawn from the absorbance values at days 1-7.

### 2.5. RNA Extraction and Quantitative Real-Time PCR

Total RNA was extracted using FastPure Cell Total RNA Isolation Kit (Vazyme, Nanjing, China) and reverse transcribed into cDNA with PrimeScript RT reagent kit (Takara, Kyoto, Japan). The cDNA amplification and detection were performed using SYBR Premix Ex Taq Kit (TaKaRa) according to the manufacturer's introduction. The primer sequences were as follows: Gapdh forward: AGGTCGGTGTGAACGGATTTG, reverse: GGGGTCGTTGATGGCAACA; Cebpb forward: CTGAGCGACGAGTACAAGAT, reverse: CTTGAACAAGTTCCGCAGG; Runx2 forward: CCTTCAAGGTTGTAGCCCTC, reverse: GGAGTAGTTCTCATCATTCCCG; Sp7 forward: GGAAAGGAGGCACAAAGAAGC, reverse: CCCCTTAGGCACTAGGAGC; Bglap2 forward: CTGACCTCACAGATCCCAAGC, reverse: TGGTCTGATAGCTCGTCACAAG. Then, the relative gene expression was normalized to the internal control (GAPDH) and analyzed with the 2^−*ΔΔ*Ct^ method.

### 2.6. Western Blot Analysis

Cells were lysated with RIPA, supplemented with a protease inhibitor cocktail (Beyotime, Shanghai, China). The protein concentration was measured with the BCA protein assay kit (Beyotime). Equal proteins were loaded onto 10% or 15% SDS-PAGE gels and then electrotransferred to a polyvinylidene difluoride membrane. The membranes were blocked in 5% nonfat milk for 2 h at room temperature and were incubated with primary antibodies at 4°C overnight. The primary antibodies were as follows: Cebpb (CST, Danvers, MA, USA), Gapdh (CST), Runx2 (CST), Hes1 (CST), Gli1 (CST), p-SMAD1/5/9 (CST), active *β*-catenin (CST), *β*-catenin (CST), Sp7 (Abcam, Cambridge, UK), p-Erk1/2 (Abcam), and Bglap2 (Santa Cruz Biotechnology, Dallas, TX, USA). The membrane was incubated with horseradish peroxidase-linked secondary antibodies for 1 h at room temperature. Finally, the blots were visualized using ECL reagents by a Bio-Rad gel imaging system. The protein intensity was quantified using Image Lab software (Version 6.0, Bio-Rad, USA).

### 2.7. Osteogenic Differentiation, Alkaline Phosphatase Assay, and Alizarin Red S Staining

mSSCs were trypsinized and replated in a 12-well plate at a concentration of 5 × 10^4^ cells per well. After being incubated in complete medium for 2 days, the medium was replaced by osteogenic induction medium (Oricell, Shanghai, China). The induction medium was changed every 3 days. The alkaline phosphatase (ALP) assay and alizarin red S (ARS) staining were conducted, respectively, on 14 days and 21 days of osteogenic induction. After being fixed for 30 min at room temperature with 10% neutral-buffered formalin, mSSCs were incubated with a BCIP/NBT solution (Beyotime, Shanghai, China) or alizarin red S solution (Beyotime) at room temperature for 10 min. ALP activities were assessed using p-nitrophenyl phosphate (pNPP, Beyotime), and the OD values were measured at 405 nm. ARS staining were quantified using 10% cetylpyridinium chloride (CPC, Beyotime) in 10 mM sodium phosphate for 15 min at room temperature, and the OD values were measured at 562 nm.

### 2.8. Animal Models

Animal researches were approved by the Institutional Ethics Committee of the East Hospital affiliated to Tongji University, School of Medicine. Three-day-old and 6-week-old male C57/BL6 mice were purchased from JSJ lab (shanghai, China). Three-day-old mice were used for mSSC isolation as above. Six healthy 6-week-old male C57/BL6 mice were randomly and equally assigned into 2 groups. The monocortical defect model was followed by the procedure previously reported [12, 28]. Briefly, the mouse was anesthetized and analgesic until there was no withdrawal reflex for surgery. Mouse was placed in the prone position, and a 1 cm incision was made at the back of the femur. A 1 mm diameter hole was made on the outside of the middle part of the femur by a 21 G needle. The hole should penetrate only one side of the femoral cortex. A total of 2 × 10^5^ mSSCs were loaded onto collagen I (Gibco, NY, USA) and incubated at 37°C for 2 h to allow collagen scaffold forming. Scaffolds were implanted into the defect sites.

### 2.9. Microcomputer Tomography (Micro-CT) Examination and Three-Point Bending Mechanical Testing

The monocortical defect model can form hard callus 3 weeks after surgery; thus, we performed micro-CT to evaluate fracture healing immediately after operation and 21 days after operation. Briefly, anesthetized mice were imaged using Skyscan 1273 (Bruker Medical, Germany) with a voltage of 70 keV, a current of 114 *µ*A, and 10.5 *µ*m isotropic resolution. The 3D reconstruction and analysis were performed with the CTAN software. The fracture defects were selected as the total volume (TV), and defect margins were located immediately after operation. The bone volume represents the volume of mineralized tissue.

Monocortical defect samples and contralateral femurs were harvested for mechanical testing using a three-point bending device (MTS Systems, MN, United States) at 21 days after operation. The femur was fixed on two support points 8 mm apart and placed in the anterior–posterior direction. The loading plate was positioned perpendicular to the fracture site during the test. The modulus of elasticity (E-modulus), ultimate load, and energy to failure were obtained and analyzed using built-in software. The biomechanical properties of the healing fractures were expressed as the percentage of the contralateral intact femur properties.

### 2.10. Immunohistochemistry

The femora were fixed in 10% neutral-buffered formalin, decalcified with EDTA Decalcified Solution, and embedded in paraffin. Sections (5 *μ*m) were cut along the long axis of samples. Antigen retrieval was conducted by mild heating (100°C) paraffin slides in EDTA Antigen Retrieval Solution (Beyotime, Shanghai, China) for 10 min. Sections were incubated with primary antibodies at 4°C overnight and HRP-linked secondary antibodies at room temperature for 1 h. The primary antibodies were as follows: Cebpb (CST, Danvers, MA, USA), Sp7 (Abcam, Cambridge, UK), and Col2*α*1 (Santa Cruz Biotechnology, Dallas, TX, USA). The fresh DAB solution was used for chromogenic detection, and hematoxylin was used for nuclear counterstaining. The stained sections were examined and photographed via microscopy at 200x magnification. Immunohistochemical analyses were reviewed and scored by 2 pathologists with over 10 years of experience: 0 (less than 10% cells were stained), 1 (10%-25% cells were stained), 2 (25%-50% cells were stained), 3 (50%-75% cells were stained), and 4 (75%-100% cells were stained).

### 2.11. Statistical Analysis

Statistical analyses were performed with GraphPad Prism 7.0 software (GraphPad, San Diego, CA, USA). Experiments were performed three times, and similar results were obtained. Results were presented as mean values ± standard deviation (SD). The statistical significance of differences between two groups was assessed using unpaired two-tailed Student's *t*-tests. The statistical significance of differences among more than two groups was assessed using one-way analysis of variance (ANOVA) with post hoc Tukey multiple comparison tests. *p* < 0.05 was considered statistically significant.

## 3. Results

### 3.1. Comprehensive Analysis of Significant TFs during Different mSSC Differentiation Stages

**Figure 1:**
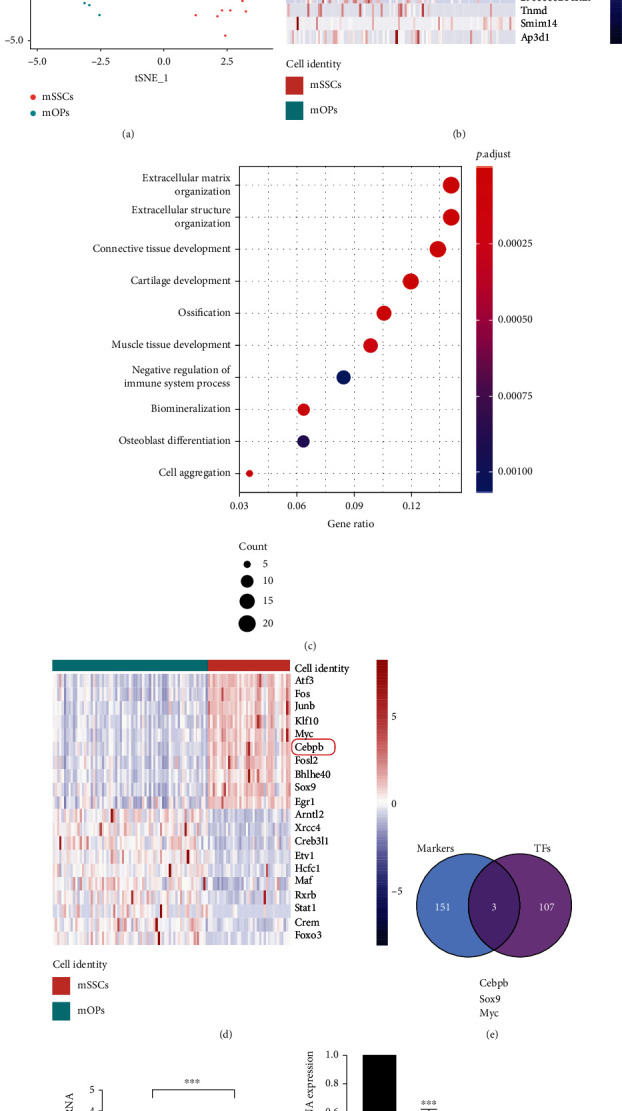
Significant TFs during different mSSC differentiation stages. (a) t-SNE analyses on scRNA-seq data of mSSCs and mOPs. (b) A heat map shows the expression of cluster markers. (c) Top 10 biological processes from GO enrichment of significant markers. (d) A heat map shows the expression of significant TFs. (e) Venn diagram of significant markers and significant TFs. (f) Comparison of normalized Cebpb expression in mSSCs and mOPs in the GSE142873 dataset. ^∗∗∗^*p* < 0.001. (g) The relative expression of Cebpb mRNA on day 0, day 7, day 14, and day 21 of mSSC osteogenic differentiation detected by qPCR. ^∗∗∗^*p* < 0.001 compared with 0 d. Abbreviations: mOPs: mouse osteoprogenitors; mSSCs: mouse skeletal stem cells; TFs: transcription factors.

To identify potential TFs regulating mSSC osteogenic differentiation, we analyzed scRNA-seq data deposited at the GEO website (GSE142873), which included mSSC and mOP clusters. Nonlinear dimensionality reduction analysis (t-SNE) suggested that these two clusters of cells could be distinguished separately (Figure 1(a)). A total of 154 significant markers were identified, and the heat map representing the top 10 markers for each cell cluster is shown in Figure 1(b). The GO analysis of markers showed that significant markers were enriched in 22 GO terms. All top 10 terms were related to mSSC differentiation, especially extracellular matrix organization (GO:0030198), ossification (GO:0001503), biomineralization (GO:0110148), and osteoblast differentiation (GO:0001649) (Figure 1(c)). To further explore TFs regulating mSSC differentiation, we analyzed the scaled data of the above datasets and constructed the regulon activity scores by SCENIC package. A total of 110 significant TFs were identified, and the heat map representing the top 10 TFs for each cell cluster is shown in Figure 1(d). As shown in Figure 1(e), the intersection of significant markers and TFs contains 3 genes: Cebpb, SOX9, and Myc. Considering that Cebpb is the most abundant gene and presented in the first 10 genes of markers and TFs, we decided to further study the role of Cebpb in mSSC differentiation. As shown in Figure 1(f), Cebpb expression was significantly higher in mSSCs than in mOPs. To further explore the role of Cebpb in different stages of mSSC osteogenic differentiation, we detected the expression of Cebpb on days 0, 7, 14, and 21 of mSSC osteogenic differentiation and found that Cebpb decreased significantly with mSSC osteogenic differentiation (Figure 1(g)), which further testify Cebpb an inhibitory role in mSSC osteogenic differentiation.

### 3.2. Isolation and Identification of mSSCs

**Figure 2:**
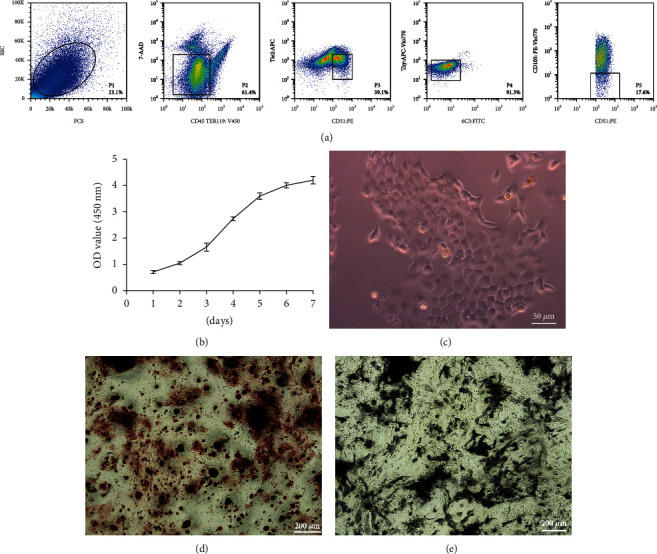
Isolation and identification of mSSCs. (a) Isolation of mSSCs. (b) Growth curve of mSSCs in vitro. (c) Colony formation of mSSCs. (d) Alizarin red S staining of mSSCs. (e) Alkaline phosphatase staining of mSSCs.

As shown in Figure 2(a), we isolated mSSCs from P3 C57/BL6 mice as previously. CD45^−^Tie2^−^Ter119^−^CD51^+^6C3^−^Thy^−^CD105^−^ cells were isolated as mSSCs, which can differentiate into mOPs and osteoblasts [27]. The cells were cultured in vitro. Growth curve during 7-day in vitro culturing suggested that the cell doubling time was approximately 2-3 days (Figure 2(b)). For the identification of mSSCs, we found it could form a colony when it is cultured in vitro (Figure 2(c)). Meanwhile, mSSCs could secrete ALP and form mineralized nodules in osteogenic differentiation by ALP assay and alizarin red S staining (Figures 2(d) and 2(e)).

### 3.3. Cebpb Knockdown Promoted mSSC Osteogenic Differentiation

**Figure 3:**
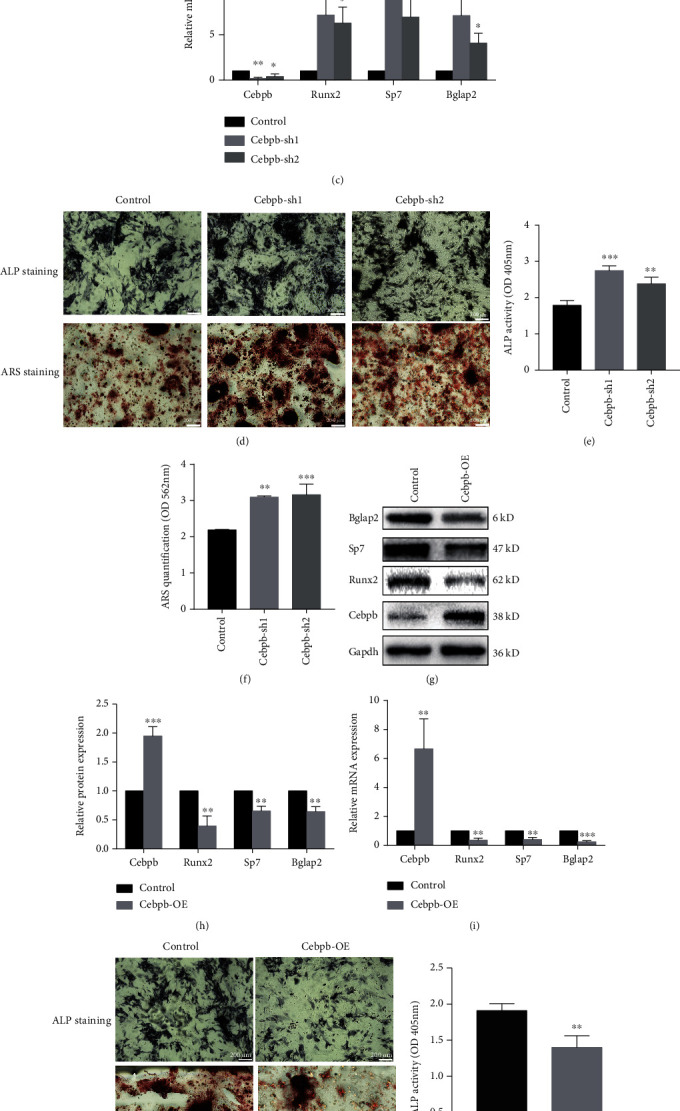
Cebpb knockdown promoted mSSC osteogenic differentiation. (a) Expression of osteogenic differentiation markers determined by western blot in the Cebpb inhibition group and the control group. (b) Quantification of result (a). (c) Expression of osteogenic differentiation markers determined by qPCR in the Cebpb inhibition group and the control group. (d) Alizarin red S staining and alkaline phosphatase staining of mSSCs in the Cebpb inhibition group and the control group. Scale bar: 200 *μ*m. (e) Quantification of alkaline phosphatase staining of result (d). (f) Quantification of alizarin red S staining of result (d). (g) Expression of osteogenic differentiation markers determined by western blot in the Cebpb overexpression group and the control group. (h) Quantification of result (g). (i) Expression of osteogenic differentiation markers determined by qPCR in the Cebpb overexpression group and the control group. (j) Alizarin red S staining and alkaline phosphatase staining of mSSCs in the Cebpb overexpression group and the control group. Scale bar: 200 *μ*m. (k) Quantification of alkaline phosphatase staining of result (j). (l) Quantification of alizarin red S staining of result (j). Cebpb-sh1: mSSCs stably expressing the Cebpb-shRNA1 sequence; Cebpb-sh2: mSSCs stably expressing the Cebpb-shRNA2 sequence; Cebpb-OE: mSSCs stably expressing the Cebpb full-length cDNA sequence. ^∗^*p* < 0.05, ^∗∗^*p* < 0.01, and ^∗∗∗^*p* < 0.001.

mSSCs were stably transfected with lentivirus containing either scramble shRNA (control) or target shRNA directed against Cebpb (Cebpb-sh1 and Cebpb-sh2). Both shRNA could knockdown Cebpb effectively in mSSCs by western blot and qPCR (Figures 3(a)–3(c)). To testify whether Cebpb knockdown could affect osteogenic differentiation, we performed the protein and mRNA expression of Runx2 and Sp7 (markers for mOPs) and Bglap2 (marker for osteoblasts) in mSSCs. As shown in Figures 3(a)–3(c), Cebpb knockdown could enhance Runx2, Sp7, and Bglap2 expression effectively. The ALP activity and ARS staining showed Cebpb knockdown could promote ALP secretion and calcium nodule formation in mSSCs (Figures 3(d)–3(f)).

To exclude any off-target effect, we introduced lentivirus containing full-length Cebpb cDNA (Cebpb-OE) or no start codon Cebpb cDNA (Control) in mSSCs. As shown in Figures 3(g)–3(i), Cebpb overexpression could inhibit the expression of Runx2, Sp7, and Bglap2. The ALP activity and ARS staining showed Cebpb overexpression could inhibit ALP secretion and calcium nodule formation in mSSCs (Figures 3(j)–3(l)). Both knockdown lentiviral vectors reached similar conclusions in the osteogenic differentiation of mSSCs, and the overexpression of Cebpb vector can inhibit the osteogenic differentiation of mSSCs, indicating that the knockdown lentiviral vector and the overexpression of Cebpb vector have no obvious off-target effects. These results suggested that Cebpb knockdown could promote mSSC osteogenic differentiation.

### 3.4. Cebpb Knockdown Activated WNT/*β*-Catenin Signaling in mSSC Osteogenic Differentiation

**Figure 4:**
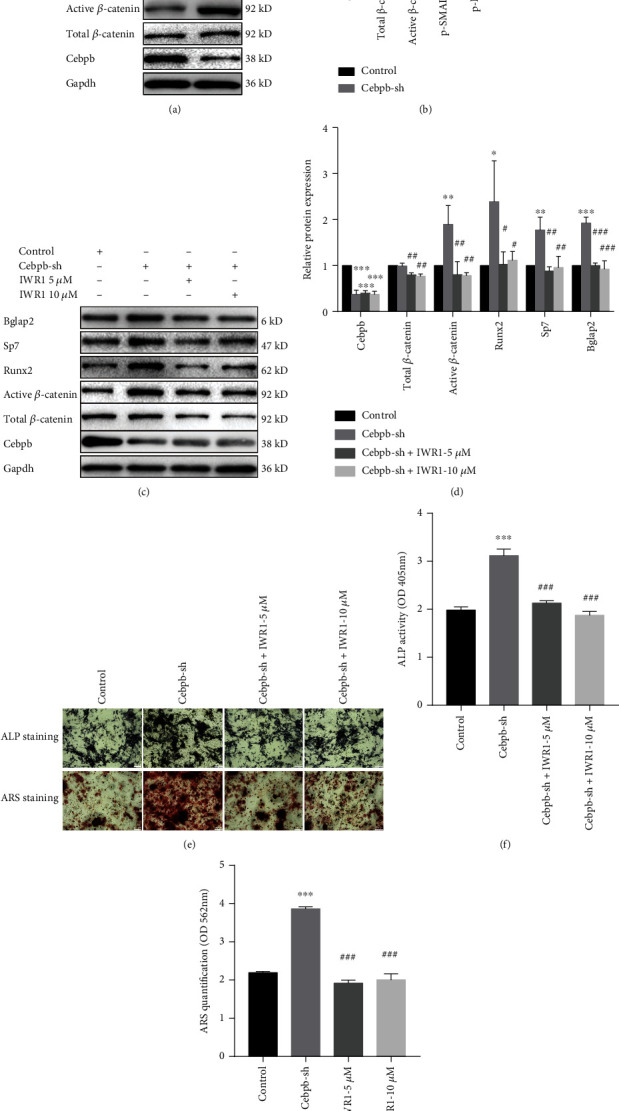
Cebpb knockdown activated WNT signaling in mSSC osteogenic differentiation. (a) Expression of osteogenic differentiation signaling proteins determined by western blot in the Cebpb inhibition group and the control group. (b) Quantification of result (a). (c) Expression of WNT signaling and osteogenic differentiation proteins determined by western blot in the control, Cebpb inhibition, and WNT signaling inhibition groups. (d) Quantification of result (c). (e) Alizarin red S staining and alkaline phosphatase staining of mSSCs in the control, Cebpb inhibition, and WNT signaling inhibition groups. Scale bar: 200 *μ*m. (f) Quantification of alizarin red S staining of result (e). (g) Quantification of alizarin red S staining of result (e). Cebpb-sh: mSSCs stably expressing the Cebpb-shRNA1 and Cebpb-shRNA2 sequence. ^∗^*p* < 0.05 compared with the control group, ^∗∗^*p* < 0.01 compared with the control group, ^∗∗∗^*p* < 0.001 compared with the control group, ^#^*p* < 0.05 compared with the Cebpb-sh group, ^##^*p* < 0.01 compared with the Cebpb-sh group, and ^###^*p* < 0.001 compared with the Cebpb-sh group. IWR1: inhibitor of WNT signaling.

To determine how Cebpb knockdown promoted mSSC osteogenic differentiation, we investigated 5 signaling pathways playing pivotal roles in bone regeneration [29]. The representative proteins are Hes1 (marker for Notch signaling), Gli1 (marker for Hedgehog signaling), p-Erk1/2 (marker for MAPK signaling), p-Smad1/5/9 (marker for BMP2 signaling), and *β*-catenin (marker for WNT/*β*-catenin signaling), respectively. The results showed that Cebpb knockdown could significantly enhance the WNT/*β*-catenin activity, but has no significant effect on the other four osteogenic-related signaling pathways in Figures 4(a) and 4(b). To further confirm the role of WNT signaling in the regulation of mSSC osteogenic differentiation by Cebpb, we added the specific WNT signaling inhibitor IWR-1 to Cebpb-sh mSSCs. The results showed that inhibiting the WNT signaling can decrease the osteogenic gene expression in Cebpb-sh mSSCs. The ALP activity and ARS staining showed WNT signaling inhibition could decrease ALP secretion and calcium nodule formation in Cebpb-sh mSSCs (Figures 4(e)–4(g)). In all, Cebpb knockdown leads to enhanced mSSC osteogenic differentiation through the WNT/*β*-catenin signaling.

### 3.5. Cebpb Knockdown Accelerated Fracture Healing

**Figure 5:**
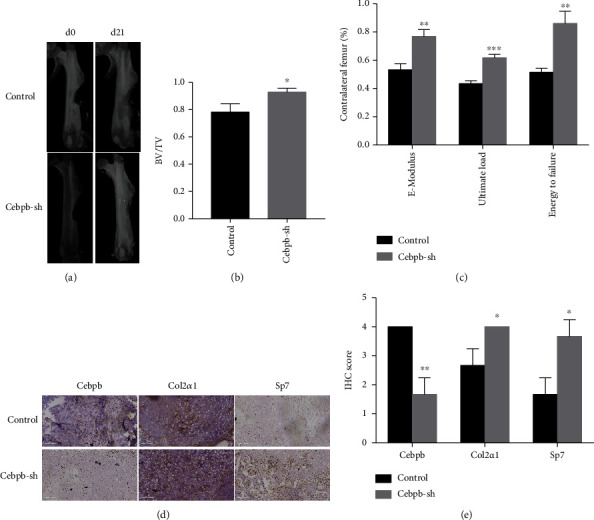
Cebpb knockdown accelerated fracture healing. (a) Representative micro-CT 3D reconstruction pictures in the control and Cebpb-sh groups. (b) Quantification of BV/TV of result (a). (c) Biomechanical test of injured femur compared with contralateral limbs. (d) Representative IHC pictures of monocortical defect tissues in the control and Cebpb-sh groups (200x). Scale bar: 100 *μ*m. (e) Quantification of IHC scores of result (d). BV: bone volume; TV: total volume. ^∗^*p* < 0.05, ^∗∗^*p* < 0.01, and ^∗∗∗^*p* < 0.001.

To determine whether Cebpb knockdown could accelerate fracture healing, Cebpb-sh mSSCs or control mSSCs and collagen I were mixed and loaded at fracture sites. At 3 weeks after fracture, the left defects were much smaller in the Cebpb knockdown group than in the control group (Figure 5(a)). The BV/TV calculated by micro-CT indicated that much more newly formed mineralized bone could be detected in the Cebpb knockdown group compared to the control group in 3 weeks after fracture (Figure 5(b)). To investigate the ultimate outcome of healing quality after Cebpb intervention, mechanical testing was performed to detect the biomechanical properties. The results showed a significant improvement in 𝐸-modulus, ultimate load, and the energy to failure in the Cebpb knockdown group after being normalized to the contralateral intact femur (Figure 5(c)). Representative sections from 2 groups stained with Cebpb, Col2*α*1, and Sp7 were shown in Figure 5(d). Much more chondrocytes and osteoblasts could be detected in the Cebpb knockdown group than the control group, which means that Cebpb knockdown may promote endochondral ossification.

## 4. Discussion

Various complications of fracture, especially nonunion, represent 5%-10% of all fractures and may seriously affect people's quality of life and increase the economic burden, which is a critical clinical challenge [2, 4]. With the increase of risk factors such as diabetes, osteoporosis, and aging, the proportion of nonunion and delayed union of fractures has increased recently [2, 4]. There are two types of nonunions, namely, atrophic and hypertrophic nonunion [29, 30]. The occurrence of hypertrophic nonunion is caused by inaccurate fixation of the fracture ends, which can be cured by refixation. Atrophic nonunion is caused by the failure of osteoblasts to form due to biological factors at the fracture sites. It cannot be cured by simple reoperation and fixation. Autograft bone transplantation, which has been over 10 decades of clinical practice, is still the gold standard for the treatment of nonunion. Although there are new technologies such as the Reamer Irrigator Aspirator to complete the extraction of bone grafts, there are still significant complications of autograft harvesting [7]. Studies have shown that the occurrence of atrophic nonunion is related to the destruction of SSCs after injury [30]. Therefore, stem cell-based therapy may be able to improve fracture healing and reduce the occurrence of atrophic nonunion.

The seminal findings of Friedenstein in 1974 showed colonic formation ability in vitro and self-renewal ability in vivo of BMSCs, named colony-forming unit-fibroblasts (CFU-Fs), which lays the foundation for SSC research [31]. SSCs with different markers located at different anatomical sites have been identified through lineage tracing and serial transplantation [14, 16–18]. The research of Chan showed the fracture leads to resting SSCs active, with increased plating efficiency, significantly greater osteogenic ability, and markedly reduced apoptotic activity [16]. Directing the fate of SSCs towards the desired lineage may provide great potential for bone maintenance and bone repair. The phenomenon that SSCs were involved in fracture healing has been testified by other researchers [18, 32, 33]. In the present study, we also testify the value of SSCs in fracture healing and suggested that SSCs can be used for nonunion treatment.

The differentiation of SSCs is affected by multiple signaling pathways, especially the canonical WNT signaling and BMP signaling [29]. The BMP signaling pathway was the first signaling pathway to be found to influence osteogenesis. BMP binds to BMP receptor to phosphorylate BMP type 1 receptor and SMAD family of proteins. The phosphorylated SMAD proteins regulate the expression of Runx2, Sp7, Bglap2s, and Col1a1, which are normally upregulated during bone formation and repair. The canonical WNT signaling plays a synergetic role with the BMP signaling. In the canonical WNT signaling, WNT ligands bind to the cell surface receptor and lead to the accumulation and dephosphorylation of *β*-catenin, enabling it to translocate to the nucleus. Active *β*-catenin associates with T cell factor (TCF) to promote transcription of BMPs and growth factors in the nucleus, facilitating SSC differentiation [34]. Depletion of *β*-catenin promotes SSCs to lipogenesis, leading to bone mass loss [35]. Our research justified that inhibition of the transcription factor, Cebpb, could accelerate SSC osteogenic differentiation by regulating canonical WNT signaling, which is in accordance with previous research.

Various TFs play a crucial role in the differentiation of BMSCs, controlling the change of cell identity [21]. Among these TFs, the most well-known ones are Runx2 and SOX9 [21, 36]. Runx2 directs BMSCs to differentiate into osteoblasts and inhibits adipogenic and chondrogenic differentiation [21]. Sox9 is an early TF of chondrogenic differentiation and controls the expression of key genes in chondrogenesis [36]. Other TFs such as Sp7, PPAR*γ*, MyoD, GATA4, and GATA6 have also been reported to play a key role in BMSC differentiation [37]. The TFs affecting SSCs have not been studied in depth, and studying these factors will bring new clinical treatment prospects and potential targets for bone repair. Cebpb contains a basic leucine zipper domain and functions as a homodimer or heterodimer. Biological processes that Cebpb regulates include embryonic development, immune, and inflammatory responses [38]. In terms of cell stemness and differentiation, Cebpb can inhibit the stemness and proliferation of cancer stem cells [39]. The role of Cebpb in the osteogenic differentiation of cells has not been reported. Our research shows that inhibiting Cebpb can promote the osteogenic differentiation of SSCs and participate in fracture healing. Considering the opposite effects of osteogenesis and adipogenesis, this finding is consistent with previous reports that Cebpb may facilitate adipocyte differentiation [40, 41].

Our research also has some limitations. Firstly, although there are multiple markers to prospectively identify SSCs, we have selected the currently widely accepted ones that have been proven to promote fracture healing through a comprehensive analysis of the literature [11, 14, 16–18, 32]. The overlapping relationship between the SSCs of this population and the SSCs marked by other markers is still uncertain [14–18, 32, 33]. Secondly, as with all preliminary animal experiments, our experiments are carried out in mouse cells and mouse fracture models. Whether the results can be extended to humans remains to be further studied. We will answer these questions in the follow-up research and will also examine the role of Cebpb in the stemness and chondrogenic differentiation of SSCs.

## 5. Conclusions

**Figure 6:**
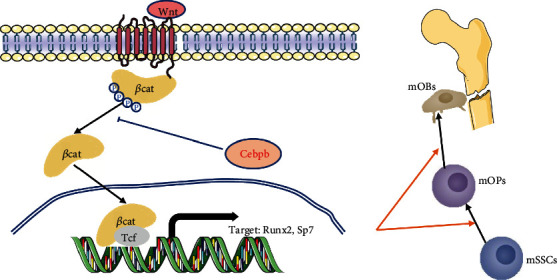
Schematic diagram of Cebpb in regulating mSSC differentiation and fracture healing through WNT/*β*-catenin signaling. *β*-cat: *β*-catenin; mSSCs: mouse skeletal stem cells; mOPs: mouse osteoprogenitors; mOBs: mouse osteoblasts.

In this study, we reanalyzed the scRNA-seq data of mSSCs and mOPs by a newly developed Seurat package and SCENIC package, concluding that Cebpb may play a key role in the osteogenic differentiation of mSSCs. We adopted FACS to sort mSSCs and carried out colony formation assay and osteogenic differentiation in vitro to confirm its identity. The roles of Cebpb in osteogenic differentiation of mSSCs are further identified through lentivirus transfection and osteogenic differentiation in vitro and in vivo. The signaling that Cebpb influenced was canonical WNT signaling in mSSC osteogenic differentiation. A schematic diagram of our results is shown in Figure 6. Cebpb knockdown can intercept its inhibitory effect on *β*-catenin activation, thereby regulating the expression of osteogenesis-related Runx2, Sp7, and Bglap2, promoting osteogenic differentiation and fracture healing. In all, we found that Cebpb regulates SSC osteogenic differentiation and fracture healing via the WNT/*β*-catenin pathway, which can be a promising target for the treatment of fracture and fracture nonunion.

## Data Availability

The datasets used and analyzed during the current study are available on GEO websites (https://www.ncbi.nlm.nih.gov/geo/query/acc.cgi?acc=GSE142873). All raw data for statistical analysis are available on the GitHub website (https://github.com/03101yly/paper_data).
